# Epidemiological and clinical features of Croatian children and adolescents with a PCR-confirmed coronavirus disease 2019: differences between the first and second epidemic wave

**DOI:** 10.3325/cmj.2020.61.491

**Published:** 2020-12

**Authors:** Nina Krajcar, Lorna Stemberger Marić, Anja Šurina, Sanja Kurečić Filipović, Vladimir Trkulja, Srđan Roglić, Goran Tešović

**Affiliations:** 1 Pediatric Infectious Diseases Department, University Hospital for Infectious Diseases “Dr. Fran Mihaljević,” Zagreb, Croatia; 2 University of Zagreb, School of Dental Medicine, Zagreb, Croatia; 3 Croatian Institute for Public Health, Zagreb, Croatia; 4 Department of Pharmacology, University of Zagreb School of Medicine, Zagreb, Croatia; 5 University of Zagreb School of Medicine, Zagreb, Croatia; Krajcar et al: Epidemiological and clinical features of Croatian children and adolescents with COVID-19

## Abstract

**Methods:**

Data on patients aged ≤19 years with a positive SARS-CoV-2 PCR test recorded in the period March 12-May 12 (first wave) and June 19-July 19, 2020 (second wave) were retrospectively analyzed. The periods were separated by several weeks with no incident cases.

**Results:**

We analyzed data on 289 children and adolescents (6.5% of all cases; incidence rate [IR] = 3.54, 95% confidence interval [CI] 3.14-3.97/million person-days), 124 in the first wave (IR = 2.27) and 165 in the second wave (IR = 6.37): IRR second/first = 2.71 (2.13-3.44). During the first wave, the incidence was highest in infants (IR = 3.48), while during the second wave it progressively increased to IR = 7.37 in 15-19-year olds. Family members were the key epidemiological contacts (72.6% cases), particularly during the first wave (95.8% vs 56.3%). Overall, 41.3% patients were asymptomatic, 25.3% in the first and 52.6% in the second wave. Age 15-19 years (vs younger) was associated with a higher (RR = 1.26, 1.02-1.54) and infection in the second wave with a lower probability (RR = 0.66, 0.53-0.81) of being symptomatic. The most common symptoms were fever, cough, and rhinorrhea. In children aged ≥7 years, headache, anosmia/ageusia, and sore throat were also recorded. Only one child suffered a severe disease. All but 18 (7.8%) children were treated only symptomatically, and all fully recovered.

**Conclusion:**

A large proportion of SARS-CoV-2 PCR-positive children/adolescents were asymptomatic. The associated disease was predominantly mild, comparably so in the first and second pandemic wave.

Since the late December 2019, coronavirus disease 2019 (COVID-19) caused by the novel severe acute respiratory syndrome coronavirus 2 (SARS-CoV-2) has spread quickly worldwide and as of early December accounts for more than 65 million cases diagnosed in more than 200 countries (1). At this point, the most affected countries in Europe are Russia, Spain, France, United Kingdom (UK), and Italy with consequently the highest mortality rates. The first case in Croatia was reported in the late February 2020, and within the next two months the infection expanded nationwide. During this first epidemic wave, Croatia was under a one-month lockdown, which rapidly decreased the disease incidence, and only a few newly diagnosed cases were reported between May 25 and June18, 2020. Easing of restrictions increased the incidence in late June, causing a second wave of COVID-19 in Croatia, with >147 000 cases reported so far (1,2).

Over the last two decades, there were two other coronavirus outbreaks. Severe acute respiratory syndrome coronavirus appeared in 2002, affecting around 8000 people, with 10% mortality. Children (4 months-17 years) accounted for <0.02% of total cases, and there was no reported death in this age group. During the outbreak of the Middle East respiratory syndrome coronavirus, around 2300 people were infected, and children (<19 years of age) were rarely affected as well (2% of total cases; 2 reported deaths) (3,4). COVID-19 has exhibited a similar epidemiological pattern. Although early reports from China, Italy, and the United States (US) suggested that children and adolescents accounted for only 1%-2% of the overall COVID-19 cases (5-7), later reports around the world indicated a higher proportions of pediatric cases, between 1%-8% (8-10). Children of all ages can be affected by SARS-CoV-2 infection, but in contrast to other respiratory viruses, they usually suffer a mild or asymptomatic infection. Compared with adults, severe infections and fatal outcomes in children are rare, and several immunopathological mechanisms could be responsible for such differences in disease severity (11). Although many studies have reviewed the features of adults with COVID-19, overall data regarding pediatric cases are scarce, and most of them are reports from China and the US, with only a few studies describing disease in children from European countries.

We aimed to describe epidemiological and clinical features of children and adolescents with COVID-19 confirmed by the polymerase chain reaction (PCR) test for SARS-CoV-2 in Croatia and to assess potential differences between the first (March-May 2020) and second (on-going) pandemic wave (June-July 2020).

## Patients and methods

In this retrospective analysis, eligible for inclusion were children and adolescents (0-19 years old) from the entire Croatia in whom a positive real-time PCR (RT-PCR) test for SARS-CoV-2 in nasopharyngeal/oropharyngeal swab samples was recorded between March 12, 2020 (the first positive PCR test result in a child in Croatia) and May 12, 2020 (the last positive PCR test result in a child during the first pandemic wave), and between June 19 (the first positive PCR test result in a child after several weeks) and July 19, 2020 (the second and still on-going pandemic wave). These two periods were separated by several weeks with virtually no incident cases. The samples were collected and processed in regional hospitals and Public Health Departments across the country. During the first wave, testing was recommended for persons with: 1) respiratory symptoms without alternate etiology and positive epidemiological criteria (travel to areas with local COVID-19 transmission/close contact with a confirmed or possible COVID-19 case within 14 days); 2) severe acute respiratory infection that cannot be explained by another etiology. Indications for testing during June/July were more extensive and referred to other, non-respiratory symptoms (eg, diarrhea/vomiting, headache), and the epidemiological criteria did not have to be present if the symptoms were COVID-19 compatible. The children were initially identified through the Croatian National Infectious Diseases Registry. Data from all patients treated in the main Croatian regional hospitals (Zagreb, Rijeka, Split, Osijek, Pula) were included. Epidemiological and clinical particulars were extracted from hospital medical records or, where appropriate, through direct contacts with the parents/guardians or adolescents (≥16 years of age) who had signed an informed consent. This study was approved by the Ethics Committees of the University Hospital for Infectious Diseases “Dr. Fran Mihaljević” and the Croatian Institute of Public Health.

### Statistical analysis

All recorded cases were used to calculate overall and age-specific incidence rates (IR) using the 2011 Croatian census data for age groups 0-19 years. Incidence rate ratios (IRR second/first wave) were used to compare the incidence rates between two pandemic waves. Epidemiological and clinical characteristics are summarized overall and by pandemic wave. Patients were classified in respect to disease severity according to the WHO criteria (12). To assess the potential association between age and pandemic wave and the probability of having a symptomatic disease, log-binomial models were fitted to the proportion of symptomatic children. We fitted frequentist (SAS 9.4 for Windows, Cary, NC, USA) and also more conservative Bayesian models (weakly informative conservative normal priors [0, 2.5, scaled to coefficients]) using rstanarm package in R (13).

## Results

**Figure 1:**
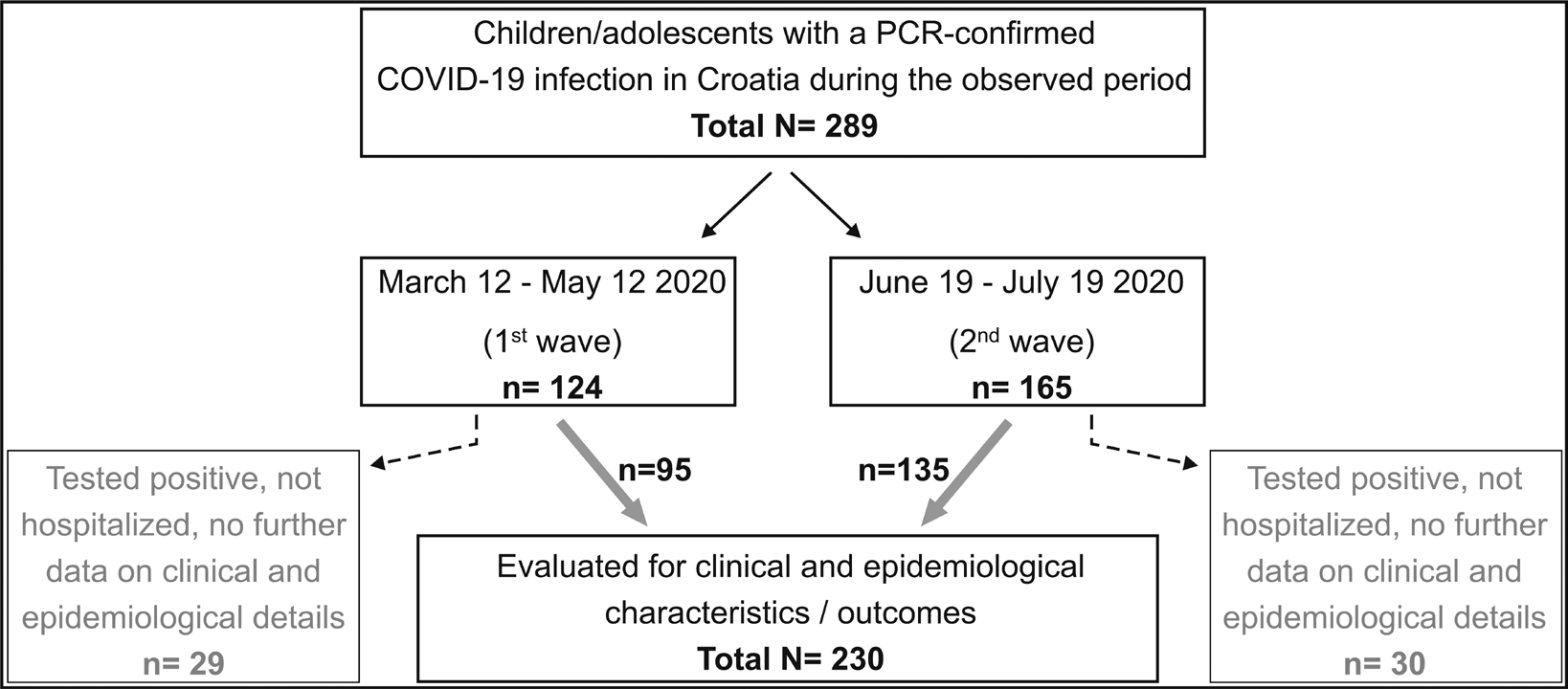
Study outline.

A total of 289 children/adolescents ≤19 years of age with a PCR-confirmed COVID-19 (6.5% of all observed COVID-19 cases in Croatia) were recorded (Figure 1): 124 during the first (5.7% of all cases during this period) and 165 during the second pandemic wave (7.3% of all cases). The overall incidence rate was 3.54 cases/million person-days, and was 2.7 times higher during the second wave (IRR = 2.71, 95%CI 2.13-3.44) (Table 1). Crude rates were practically identical to the rates age-standardized to the Standard European Population (Table 1). Overall IRs were similar across age groups (Table 1), but during the first wave IR was the highest in infants, while in the second wave it progressively increased to 7.37/million person-days in 15-19-year-olds (Table 1). Of the 289 recorded children, data on 59 (20.4%) were not available for a more detailed evaluation (Figure 1): they were not hospitalized before or within 30 days after the PCR testing, and their medical histories could not be traced. Although with some uncertainty, it is reasonable to conclude that they suffered only mild symptoms, if any at all. Considering the remaining 230 patients, family members were the main source of infection (72.6%), more so in the first wave (95.8%) than in the second wave (56.3%) (Table 2). Other likely sources of infection were social gatherings, preschools/daycare centers, and after-school activities (1/95 children in the first period vs 30.4% in the second) (Table 2). The affected children were otherwise healthy, with 16.1% suffering from pre-existing conditions, mostly asthma and recurrent bronchial spasms or pneumonia (Table 2). Apart from one newborn, all had a history of Bacillus Calmette-Guérin (BCG) vaccination. A considerable proportion of children (41.3% overall, 25.3% and 52.6% in the first and the second wave, respectively) had no symptoms before and up to four weeks after testing, hence were considered asymptomatic (Table 3). The most common symptoms were fever (body temperature >37 °C) (47.0%), which was usually low-grade (the median peak fever was 38 °C and median duration was 2 days), cough (16.5%), and runny nose (11.3%) (Table 3). Gastro-intestinal symptoms were rare (Table 3), and only 6 patients (2.6%) where tachypneic or dyspneic at a particular point in time and 5 suffered conjunctivitis (2.2%) (Table 3). Skin changes were present in only 2 patients: urticaria and maculoapapular rash. Febrile seizures were reported in one, previously healthy, toddler, while all the other children did not have any symptom of central nervous system involvement.

**Table 1:** Incidence rates (IR/1 000 000 person days of observation) of children/adolescents (age 0-19 years) with a positive polymerase chain reaction (PCR) test for SARS-CoV-2 virus during the first (March 12-May 12) and second wave (June 19-July 19) of coronavirus disease-19 pandemic in Croatia. All estimates are presented with 95% confidence intervals

	Total	First wave	Second wave	Incidence rate ratio second/first
Observed period, days	91	61	30	—
Number with +PCR	289	124	165	—
Crude IR	3.54 (3.15-3.97)	2.27 (1.88-2.70)	6.13 (5.23-7.14)	2.71 (2.13-3.44)
Crude by age in years (n, IR)				
Up to 1	15, 3.89 (2.18-6.41)	9, 3.48 (1.59-6.61)	6, 4.72 (1.73-10.3)	1.36 (0.40-4.26)
1 to 4	50, 3.22 (2.39-4.25)	22, 2.12 (1.33-3.21)	28, 5.48 (3.64-7.92)	2.59 (1.43-4.75)
5 to 9	66, 3.55 (2.74-4.52)	27, 2.17 (1.43-3.15)	39, 6.36 (4.52-8.70)	2.94 (1.75-4.99)
10 to 14	67, 3.13 (2.42-3.97)	29, 2.02 (1.35-2.90)	38, 5.38 (3.81-7.38)	2.66 (1.60-4.48)
15 to 19	91, 4.10 (3.30-5.03)	37, 2.48 (1.75-3.42)	54, 7.37 (5.54-9.62)	2.97 (1.92-4.64)
Standardized IR EU*	3.53 (3.13-3.97)	2.26 (1.88-2.70)	6.13 (5.23-7.15)	—

*Age-standardization to Standard European Population (EU-27 plus EFTA 2011-2030 population) (*https://seer.cancer.gov/stdpopulations/world.who.html,* accessed October 3, 2020)

**Table 2:** Epidemiological characteristics of children with a positive polymerase chain reaction (PCR) test for SARS-COV-2 during the first (March 12-May 12) and second (June 19-July 19) pandemic wave in Croatia. Data are count (%) if not otherwise stated

	Total	First wave	Second wave
N	230	95	135
Girls	131 (56.9)	54 (56.8)	77 (57.0)
Age in years (median, interquartile range, range)	10.0 (4.6-15.7; 0.02-19)	8.1 (3.2-15.4; 0.2-19.0)	11.8 (6.3-15.8; 0.02-18.9)
up to 1	14 (6.1)	9 (9.5)	5 (3.7)
1-4	44 (19.1)	21 (22.1)	23 (17.0)
5-9	54 (23.5)	22 (23.2)	32 (23.7)
10-14	51 (22.2)	19 (20.0)	32 (23.7)
15 19	67 (29.1)	24 (25.3)	43 (31.9)
Imported infection	11 (4.8)	9 (9.5)	2 (1.5)
Infection source: family/other/unknown	167 (72.6)/42 (18.3)/21 (9.1)	91 (95.8)/1/3	76 (56.3)/41 (30.4)/18 (13.3)
First symptomatic person			
family members	165 (71.7)	87 (91.6)	78 (57.8)
friends/other contacts	8 (3.5)	2 (2.1)	6 (4.4)
child (patient)	34 (14.8)	1 (1.0)	33 (24.4)
unknown	23 (10.0)	5 (5.3)	18 (13.3)
Children with comorbidities	37 (16.1)	21 (22.1)	16 (11.8)
asthma/recurrent spasms/pneumonia	14	7	7
allergic rhinitis/recurrent otitis media	6	4	2
rheumatologic diseases	4	2	2
any other	13	8	5

**Table 3:** Clinical characteristics of children with a positive polymerase chain reaction (PCR) test for SARS-COV-2 during the first (March 12-May 12) and second (June 19-July 19) pandemic wave in Croatia. Data are count (%) if not otherwise stated

	Total (N = 230)	First wave (n = 95)	Second wave (n = 135)
No symptoms/any symptoms	95 (41.3)/135 (58.7)	24 (25.3)/71 (74.7)	71 (52.6)/64 (47.4)
Fever/duration (days; median, range)	108 (47.0)/2 (1-14)	63 (66.3)/2 (1-14)	45 (33.3)/2 (1-8)
Peak fever (°C; median, range)	38.0 (37.2-40.0)	38.0 (37.2-40)	38.0 (37.2-39.8)
Cough	38 (16.5)	22 (23.2)	16 (11.9)
Runny nose	26 (11.3)	14 (14.7)	12 (8.9)
General infective syndrome	26 (11.3)	23 (24.2)	3 (2.2)
Vomiting	8 (3.5)	5 (5.3)	3 (2.2)
Diarrhea	7 (3.0)	4 (4.2)	3 (2.2)
Tachypnea/dyspnea	6 (2.6)	5 (5.3)	1 (0.7)
Conjunctivitis	5 (2.2)	1 (1.0)	4 (3.0)
Skin changes	2 (0.8)	1 (1.0) (urticarial)	1 (0.7) (maculopapular)
Febrile convulsions	1 (0.4)	1 (1.0)	0
Laryngitis	1 (0.4)	1 (1.0)	0
Considering children ≥7 years of age			
headache	29/151 (19.2)	15/52 (28.8)	14/95 (14.7)
anosmia/ageusia	27/151 (17.9)	13/52 (25.0)	14/95 (14.7)
sore throat	17/151 (11.3)	10/52 (19.2)	7/95 (7.4)
chest or abdominal pain	7/151 (4.6)	5/52 (9.6)	2/95 (2.1)
x-ray normal/not done/pneumonia	1/227 (98.7)/2	1 (1.1)/94 (98.9)/0	0/133 (98.5)/2 (1.5)
Hospitalized due to disease severity	2 (6 days, 11 days)	0	2 (6 days, 11 days)
Hospitalized-epidemiological reasons	9 (3.9) (7-21 days)	8 (7-21 days)	1 (9 days)
Disease severity WHO criteria			
mild disease	133/135 symptomatic	71/71 symptomatic	62/64 symptomatic
pneumonia	1	0	1/64 symptomatic
severe pneumonia	1	0	1/64 symptomatic
acute respiratory distress syndrome, sepsis, septic shock	0	0	0
Treatment apart from symptomatic	18 (7.8)	11 (11.6)	7 (5.2)
Azithromycin	8	2	6
Inhaled anti-asthmatics	4	2	2
Systemic corticosteroids	2	1	1
Inhaled epinephrine	1	1	0
Oseltamivir	1	1	0
LMWH	1	0	1
Penicillins/cephalosporins	8	6	2
Complications/sequels	0	0	0

In children ≥7 years of age, headache (19.2%), anosmia/ageusia (17.9%), and sore throat (11.3%) were also observed (Table 3). Symptomatic children experienced almost exclusively a mild disease (133/135) (Table 3). Only 2 children were hospitalized for medical reasons (Table 2), one of whom suffered pneumonia (“moderate disease”) and the other severe pneumonia: a 16-year old girl with obesity and a history of behavioral disorder was admitted to the ICU and was treated with oxygen therapy, supportive measures, anticoagulants, dexamethasone, and antibiotics (azithromycin and ceftriaxone) for suspected bacterial coinfection. There were no cases of confirmed bacterial coinfection, sepsis, acute respiratory distress syndrome, or multisystem inflammatory syndrome in children. Only 18 children (7.8%) received treatments other than symptomatic, and all fully recovered (Table 3).

There was a trend of higher probability of being symptomatic with older age, regardless of the pre-existing comorbidity (Table 4). With further adjustment for the observed period, the probability of being symptomatic was higher in 15-19-year-olds than in younger age groups (RR = 1.26, 1.02-1.54) and lower in children affected in the second wave (RR = 0.66, 0.53-0.81) (Table 5).

**Table 4:** Distribution of children with “any symptom” across age groups and in respect to comorbidity. Data are n/N (%)

	Comorbidity			
Age, years	none	respiratory/rheumatic	any other	Total
Up to 1	8/13 (61.5)	—	1/1 (100)	9/14 (64.3)
1-4	24/37 (64.9)	2/4 (50.0)	0/3 (0)	26/44 (59.1)
5-9	18/42 (42.9)	4/8 (50.0)	2/4 (50.0)	24/54 (44.4)
10-14	25/46 (54.4)	4/5 (80.0)	—	29/51 (56.9)
15-19	36/55 (64.5)	6/7 (85.7)	5/5 (100)	47/67 (70.1)
Total	111/193 (57.5)	16/24 (66.6)	8/13 (61.5)	135/230 (58.7)

**Table 5:** Summary of multivariate analysis of probability of being symptomatic*

	Frequentist		Bayesian		
	RR (95%CI)	P	RR (95%CrI)	probabilities	BF
Age 15-19 vs younger	1.26 (1.02-1.54)	0.030	1.21 (1.00-1.48)	P(RR)>1.0 = 97.2%	7.7
Second wave	0.66 (0.53-0.81)	<0.001	0.66 (0.53-0.82)	P(RR)<1.0 = 99.9%	16.6
Any comorbidity	1.19 (0.88-1.61)	0.249	1.02 (0.86-1.15)	P(RR)>1.0 = 61.6%	0.019

*RR – risk ratio; BF– Bayes factor; CrI – credible interval.

Testing (negative findings) for SARS-CoV-2 was repeated in 93/230 (40.4%) children (61.1% vs 25.9% in the first and second wave, respectively), with a time lag ranging between 4 and 63 days.

## Discussion

The present data are in line with reports from other countries suggesting that children and adolescents represent only a small proportion of COVID-19 patients. The median age in the present study was higher than reported in studies from Italy (3.3 years) and UK (4.6 years) but similar to that reported in China (6-7 years) and North America (11 years) (14-17). The present study is specific in that we collected nationwide data through a network of Public Health Departments and hospitals designated for monitoring of the pandemic and patient treatment. The epidemiological network consists of 21 regional Public Health Departments (in 21 administrative units, Counties), with rather extensive testing whenever indicated by epidemiological circumstances. As a consequence of broad testing, the estimated incidence rates could be reasonably close to the actual situation, although the important question regarding the number of incident cases (eg, asymptomatic) that were missed remains unanswered. The incidence rates suggest that in the overall observed period children/adolescents of different age were similarly affected. However, there appeared several considerable differences between the two periods: a) in the first wave the IRS were highest in newborns when compared with other age groups; b) in the second wave there was a clearly higher incidence overall and in each age group; c) in the second wave the incidence increased with older age. In part, the discrepancies are likely due to the fact that the first wave included a one-month period of a complete lockdown (closing of preschools/schools/universities/public transport, restrictions on public gatherings, international and intra- country travel, closing of all-but-essential workplaces, case isolation and home quarantine for close contacts, comprehensive contact tracing), followed by virtually no incident cases between May 25 and June 18, 2020, which led to gradual easing of restrictions. The second wave (June 19-July 19), on the other hand, was characterized by considerably relaxed epidemiological measures (reopening of preschools/workplaces, easing restrictions on public gatherings and travel, but case isolation, home quarantine for close contacts, and comprehensive contact tracing remained the same). Another possible source of the differences might be the fact that in the second wave more extensive PCR testing was undertaken (ie, broader epidemiological indications). The rise in incident cases and the observed difference in age-specific rates coincides also with the differences in the most likely transmission settings: in the first wave practically all patients acquired the infection from their family members (no cases of health care-acquired infections), while in the second wave a considerable proportion of children acquired SARS-CoV-2 outside of home (possibly from other adults or children in pre-schools and daycares). The role of children and adolescents in the context of household and community transmission is currently in the focus of intense discussions because it impacts social and economic issues and affects the reopening of schools. A European multicenter study conducted during the initial peak of COVID-19 pandemic showed that the most common source of infection in children was a parent or a sibling (60%) (14). Another study suggested that children mainly acquired the infection from an adult contact, and that secondary transmission from children was low (18). Only a few case reports have suggested transmission from children to other family members and, a relationship between school closures and transmission dynamics has not been conclusively proven (19-22).

It has been suggested that the viral spread could be affected by SARS-CoV-2 mutations. Currently, the globally predominant strain is the D614G variant, which has replaced the D614 strain (the Wuhan strain), the dominant strain in the early stage of the pandemic (23). The transformation from D614 to D614G started in Europe during March. D614G then spread to North America and the rest of the world, accounting for 78% of global sequences by the end of May 2020. While D614G results in a higher viral load and is more contagious than D614, currently there is no evidence that the infection with this variant would result in a more severe disease (23-25). Croatia has obtained whole-genome sequences for only a small number of SARS-CoV-2 isolates from patients during March and April 2020 and deposited them in GISAID (the main SARS-CoV-2 genomes open-source database). Only one strain contained the D614G variant (26,27). It was detected in a sample collected in Dubrovnik-Neretva County in late March, while other strains from different Croatian counties did not contain this mutation. Generally, the number of the presented cases, overall and by the observed period, is relatively limited, which precluded any meaningful comparisons across Croatian counties, which largely differ in population: most contributed just a few or no cases, overall or by the observed period. In the first wave, IR (age-adjusted to Croatian population) in the Dubrovnik-Neretva County was 4.18 (1.68-8.65) cases/million person-days, and it was 1.23 (0.03-6.91) in the second period. However, there were only 7 cases in the first and one case in the second period – hence, the estimated difference between the two rates (IRR = 3.40, 95%CI 0.46-141) is extremely imprecise, and data do not provide a basis for any assumptions about the relevance of the isolated D614G strain.

A large proportion of PCR-positive children/adolescents were asymptomatic: 41.3% overall, 25.3% in the first and 52.6% in the second wave, and there appeared a mild but clear trend of higher probability of being symptomatic with older age. This prevalence of asymptomatic children is considerably higher than reported in different European countries, China, Republic of Korea, Iran, and the US (in the range between 4.0% and 28.0%) (14,28-34). In this respect, the following should be noted: a) we adhered to the WHO and the European Centre for Disease Prevention and Control definition of a “COVID-19 patient” as a subject with a positive PCR test regardless of the presence of symptoms; b) the present proportions might not be exactly correct, since 59/289 of the recorded PCR-positive subjects could not have been reliably evaluated for the presence of symptoms. However, even if symptomatic, they most likely suffered a mild(er) disease and were not hospitalized; c) the designation of “asymptomatic” was based on the evaluation over a period that extended from around 7 days before the PCR test to 30 days after testing (so, no misclassification was likely); d) most of the published reports were focused on hospitalized children or those who were admitted to hospital at least transiently, while the present results pertain to children/adolescents who were tested even when considered a relatively remote contacts of other “positive” or symptomatic people. The latter notion could induce speculation about the actual positive predictive values of different PCR tests (ie, specificity in clinical settings), relevance of the viral load, and other topics that are far beyond the scope of the present work.

Practically all symptomatic children (133/135) experienced a mild disease – fever and respiratory tract infection symptoms without pneumonia, which is discordant with the suggested (higher) proportion of children with a moderate (39%) disease in a recent systematic review embracing 1475 children (32). The proportions of children with severe (2%) and critical illness (0.7%) were comparable with those in our study. The present data are also in disagreement with the suggested higher probability of a severe disease in infants (<12 months of age) (14,35-42). Furthermore, gastrointestinal symptoms were less common in the present study as compared with other reports (5%-7% or 10%-35%) (14-16,43). In a way, this is in agreement with the suggested higher prevalence of gastrointestinal symptoms in children with a more severe COVID-19 (44).

A number of hypotheses have been proposed to explain the usually mild or asymptomatic SARS-CoV-2 infection in children and young people. Possible reasons include the presence of cross-reactive antibodies due to frequent contacts with seasonal coronaviruses, different expression of angiotensin-converting enzyme 2 across age groups, recent vaccinations (BCG) that produce broad protection against viral infection and sepsis, and more active innate immune response compared with adults (45). BCG vaccination has been suggested to be associated with reduced COVID-19 morbidity and mortality (46-48). Croatia has been implementing universal BCG vaccination since 1948 and has high BCG immunization coverage, which could attenuate the severity of COVID-19 in our citizens, especially among children, who are the most recently vaccinated population. However, the discrepancy between the proportions of asymptomatic children/adolescents in the first and the second wave is difficult to explain. Apart from the more extensive testing during the second wave, no other potential explanation seems reasonable. Although it has been anecdotally (but without any sound data) suggested that with higher ambient temperature the clinical presentation of COVID-19 could be milder, the present data provide no grounds for speculations of this kind: the two observed periods were characterized by substantially different epidemiological circumstances. Further studies on viral loads could possibly explain a lower incidence during hot periods of the year.

The present analysis is limited by a relatively small sample and a lack of detailed epidemiological and clinical data for 59/289 PCR-positive participants. They were not hospitalized in regional hospitals, and we were unable to establish a direct contact with consenting parents/caregivers or primary care physicians. It seems reasonable to assume it unlikely that any clinically relevant form of the disease remained unreported/unrecorded – hence, they probably suffered only mild symptoms or were asymptomatic.

In conclusion, the present data suggest that COVID-19 in Croatian children and adolescents results in a mild and often asymptomatic disease. Critical disease forms and deaths in this age group in our population have not been reported yet.
